# Reply to: An approach to the resolution of the dispute on collective atomic interactions

**DOI:** 10.1038/s41467-024-54553-y

**Published:** 2024-11-30

**Authors:** Nuno A. G. Bandeira, Ángel Martín Pendás, Cina Foroutan-Nejad

**Affiliations:** 1 Biosystems and Integrative Sciences Institute (BioISI)- Departamento de Química e Bioquímica, Faculdade de Ciências Universidade de Lisboa, Lisboa, Portugal; 2 Dpto. Química Física y Analítica. Universidad de Oviedo, Oviedo, Spain; 3 Institute of Organic Chemistry, Polish Academy of Sciences, Warsaw, Poland

**Keywords:** Method development, Structure prediction, Computational chemistry, Chemical bonding

**replying to** P. Polestshuk *Nature Communications* 10.1038/s41467-024-54552-z (2024)

In a recent article^1^, Polestshuk critiqued our papers on collective interactions^2,3^. Collective interactions form between metallic ions and molecules/ions with general formula AX_3_ where X is an electron-rich atom or group compared to relatively electron-poor or electropositive, A. Polestshuk’s paper has three main points: (I) Choosing proper partitioning model, (II) reassessing our claim regarding the existence of collective interactions, and (III) the relevance of energy decomposition analyses and introducing a new energy decomposition analysis (EDA).

In the first section, Polestshuk legitimately points out that our original methodology, the not-so-commonly employed interacting quantum atoms (IQA)^4^, is sensitive to the selection of atomic partitions. Having been aware of this fact and to examine the sensitivity of our results to the choice of partitioning method we utilized both Bader’s quantum theory of atoms in molecules^5^ (QTAIM) and Becke’s fuzzy atom partitioning^6^. It is known that IQA results in these two limiting scenarios explore the variability of the method to the choice of atomic partition. While the former method uses atoms with sharp boundaries, the latter employs atoms with no distinct boundaries, that interpenetrate each other. The results of these fundamentally different approaches were gathered under a section entitled “To what extent the ICI (interaction collectivity index) values are sensitive to the nature of the used atomic basins?” We demonstrated that despite expected quantitative differences, both QTAIM and fuzzy atom partitioning conclude that collective interactions are real. A considerable part of Polestshuk’s article is devoted to criticizing the QTAIM partitioning, and the atomic charges derived from this method. In this regard, we point out two issues. (1) Atomic charges are not observable, and their magnitude is always model-based. (2) The role of QTAIM charges or other charge-partitioning schemes is in many ways comparable to that played by total energies in density functional theory (DFT). DFT energies are not exact, but they are useful when compared with other energies at the same level of theory. Similarly, an atom’s charge makes sense when compared with the charges of the same atom in different molecules using the same partitioning methodology. In this regard, changes in QTAIM charges behave similarly to those provided by other methods^7^.

Without leaving the IQA scheme, Polestshuk used a polyhedral (or Voronoi) atomic partitioning based on a seemingly cumbersome selection of atomic, ionic and/or covalent radii. This approach suffers from two flaws: First, in the case of LiCF_3_ Polestshuk selected an ionic radius for Li and then covalent ones for C and F, while in the end, he concludes that the Li and carbon are covalently bonded. Based on that account it seems that the radius is selected to reach the conclusion that is consistent with the author’s presumptions. Experimental ionic radii^8^ also vary depending on the coordination number of the ion and whether the experimental technique measuring it is X-ray diffraction or infrared spectroscopy. Nevertheless, whatever values one chooses they should be chosen consistently. This arbitrariness and the fact that IQA results do depend on the choice of atoms will certainly provide a spectrum of allegedly correct results, while both QTAIM and fuzzy atoms can be determined free from this arbitrariness.

Utilizing his partitioning Polestshuk came to the same conclusion as we did. Namely, he concluded that i-LiCF_3_ is stabilized via collective interactions and computed a comparable ICI value for the interaction between Li and CF_3_. Similarly, he concluded that p-LiCF_3_ is stabilized via a classical interaction. These conclusions are in line with our qualitative observations based on much simpler electrostatic potential maps^9^ and our bond dissociation energy analysis based on coupled-cluster/Hartree-Fock comparison for collective versus one-center interactions^10^.

Polestshuk took the opportunity to introduce and examine a new EDA analysis in his recent Matters Arising. We refrain from commenting on this approach as it is a new, unreviewed method, although we feel that it is similar to one presented already fifteen years ago^11^. However, we seize the opportunity to present the results of alternative charge and energy decomposition analyses on the same systems to prove that even conventional fragment partitioning confirms the existence of collective interactions if employed properly.

Firstly, we employed the Local Energy Decomposition (LED) scheme proposed by Schneider and colleagues for assessing the nature of bonding^12^. The results are expounded in Table 1 (below). The first aspect that stands out is that the additional stability of the inverted isomer, i-LiCF_3_, respective to its pyramidal isomer, p-LiCF_3_, is entirely due to dynamic correlation. The energy difference at the Hartree-Fock (HF) level is 2.8 kcal mol^−1^ in favor of p-LiCF_3_.

**Table 1:** Local energy decomposition (LED) energy terms in atomic units for both the inverted and pyramidal LiCF_3_ adducts

LED (a.u.) terms and CT direction	i-LiCF_3_	p-LiCF_3_
Sum of Intra-fragment HF energy	−343.436608944	−343.419845318
HF Electrostatic (1,2) term	−0.316668278131	−0.337008629
HF Exchange (1,2) term	−0.006408551785	−0.007321516
Total HF energy	−343.759685774	−343.764175463
CCSD CF_3_ correlation relaxation	−0.902867192	−0.909311498
CCSD Li correlation relaxation	−0.034172267	−0.034068353
CCSD CF_3_→Li charge transfers	−0.020236877 **(−12.7** **kcal** **mol**^**−1**^**)**	−0.009165010 **(−5.8** **kcal** **mol**^**−1**^**)**
CCSD Li→ CF_3_ charge transfers	−0.000092401	−0.000075675
CCSD Dispersion (1,2)	−0.001363194	−0.000865583
Weak pairs correlation	−0.000597666	−0.000687662
Total CCSD correlation	−0.979009984	−0.954098756
Triples term	−0.029262916	−0.027922324
CCSD(T) correlation	−0.988592462	−0.982021080
Total Energy	−344.749126257	−344.746965809
CF_3_→Li CT	0.1993 e^−^	0.1239 e^−^
Overlap population r	−0.0324 e^−^	−0.0503 e^−^
Donor→ acceptor AOs	Largest contributors to CF_3_→Li CT	
F: 2*p*_z_ → Li: 2*p*_z_	HOMO-8	
	0.0407 e^−^ (20% of net CT)	
F: 2*s* → Li: 2*p*_z_	HOMO-11	
	0.0280 e^−^ (14% net CT)	—
F: 2*p*_x_ → Li: 2*p*_y_	HOMO-7	
	0.0229 e^−^ (11% of net CT)	
F: 2*p*_y_ → Li: 2*p*_x_	HOMO-6	
	0.0229 e^−^ (11% of net CT)	
C: 2*p*_z_ → Li: 2*p*_z_	—	HOMO
		0.0934 e^−^ (76% of net CT)

It is worth noting that dispersion in wavefunction theory is defined on the basis of single excitations (one hole and one particle) of the reference wavefunction within each fragment (genuine dispersion) and across fragments (exchange dispersion). The energy values are reported in atomic units unless otherwise stated. Generalized charge decomposition analysis (GCDA) values of both LiCF_3_ adducts indicating the relevant CCSD natural orbitals responsible for the inter-fragment charge transfer. Note that HF in the table stands for Hartree-Fock, CCSD(T) denotes coupled cluster single, double, and (triple) level of theory, CT and AO represent charge transfer and atomic orbitals in turn.

The values in bold represent the same energy term but in kcal/mol.

Owing to the localized pair natural orbitals the energy terms pertaining to the relaxation effects by the excitation operators can be assigned to either inter- or intra-fragment classes. The terms for intra-fragment excitations do not differ much but already they tend to benefit i-LiCF_3_ slightly. The most significant difference between p-LiCF_3_ and i-LiCF_3_ lies with the CF_3_→Li charge transfers where the energy difference is almost double (5.8 vs 12.7 kcal mol^−1^). Besides, i-LiCF_3_ benefits from more interfragment dispersion compared to p-LiCF_3_. The triples perturbative energy term does not differentiate the two isomers significantly and its addition maintains the CCSD correlation picture.

To unveil the main orbital interactions characterizing the chemical bond the generalized charge decomposition analysis^13,14^ (GCDA) was performed (Table 1 and Fig. 1). The downside of this approach is that fragment charge assignment and separate calculations for each fragment are required. The fairer partition is the ionic one (Li^+^ + CF_3_^−^) and it restrains excessive polarization contributions (see Supplementary Information). The net CT is almost doubled in i-LiCF_3_ compared to p-LiCF_3_. This is consistent with the added stabilization of i-LiCF_3_. At this stage, we refrain from categorizing the bonds with labels such as ‘covalent’ or ‘ionic’, it is however sufficient to demonstrate a stronger level of engagement in the interactions between Li and F than between Li···C.

**Fig. 1:**
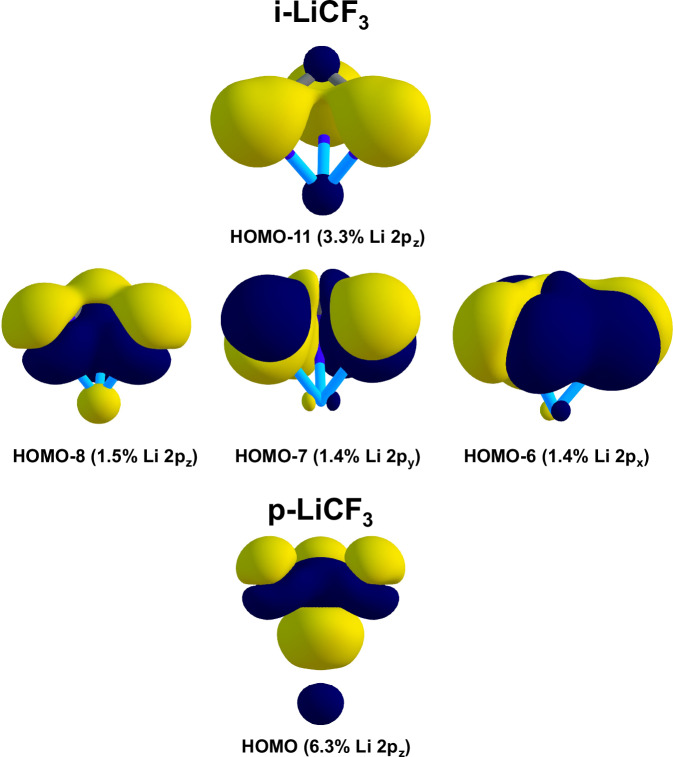
CCSD natural orbitals showing the main Löwdin contributions to donor–acceptor interaction processes in i-LiCF_3_ (top) and LiCF_3_ (bottom) as shown in Table 1.

The underlying reason for this is demonstrable through the most relevant contributions of the CCSD natural orbitals to the net electron transfer. In the case of i-LiCF_3_ there is a shared involvement of the HOMO-8, HOMO-7 and HOMO-6 that correspond essentially to the fluorine lone and bonding pairs (2*p*_x_, 2*p*_y_,2*p*_z_) and within the CF_3_ moiety admixed with an Li AO with partial 2p character. This naturally corresponds to a donor-acceptor interaction. The only striking feature is that this does not take place in the frontier orbital region. This is typical of a charge-controlled adduct that is dominant in hard acid-base chemistry^15^.

The pyramidal isomer however shows its largest (and nearly exclusive) CT contribution in the HOMO where lithium is compelled to interact with a softer Lewis base, carbon.

The novel results yield a more complete picture of the chemical bond in both i-LiCF_3_ and p-LiCF_3_. They show two main aspects:

1. Preference for ‘collective bonding’ by tripodal ligands is a dynamic correlation phenomenon at least for the LiCF_3_ case. Further studies extended to other Lewis acid-base pairs would clarify a hypothetical generalization.
2. Charge transfer is a dominant and indeed necessary feature for the stabilization of the i-LiCF_3_ isomer.

## Supplementary information

Supplementary Information

## Data Availability

The computational methods, coordinates of the molecules employed for the analyses, and the details of charge and energy partitioning in this study are provided in the Supplementary Information.

## References

[1] Polestshuk, P. An approach to the resolution of dispute on collective atomic interactions. *Nat. Commun*. 10.1038/s41467-024-54552-z (2024). 10.1038/s41467-024-54552-z PMC11608355 39616190

[2] Sowlati-Hashjin, S. et al. Collective interactions among organometallics are exotic bonds hidden on lab shelves. *Nat. Commun.* **13**, 2069 (2022). 35440588 10.1038/s41467-022-29504-0 PMC9018958

[3] Šadek, V. et al. Reply to: On the existence of collective interactions reinforcing the metal-ligand bond in organometallic compounds. *Nat. Commun.* **14**, 3873 (2023). 37400455 10.1038/s41467-023-39504-3 PMC10317954

[4] Blanco, M. A., Martín Pendás, A. & Francisco, E. Interacting quantum atoms: a correlated energy decomposition scheme based on the quantum theory of atoms in molecules. *J. Chem. Theory Comput.* **1**, 1096–1109 (2005). 26631653 10.1021/ct0501093

[5] Bader, R. F. W. *Atoms in Molecules: A Quantum Theory* (Clarendon Press, Oxford; New York, 1990).

[6] Becke, A. D. A multicenter numerical integration scheme for polyatomic molecules. *J. Chem. Phys.* **88**, 2547–2553 (1988).

[7] Cho, M. et al. The atomic partial charges arboretum: trying to see the forest for the trees. *ChemPhysChem* **21**, 688–696 (2020). 32052532 10.1002/cphc.202000040 PMC7317385

[8] Shannon, R. D. Revised effective ionic radii and systematic studies of interatomic distances in halides and chalcogenides. *Acta Crystallogr. A* **32**, 751–767 (1976).

[9] Pino-Rios, R., Báez-Grez, R. & Foroutan-Nejad, C. Anti-electrostatic cation⋯π-hole and cation⋯lp-hole interactions are stabilized via collective interactions. *Chem. Commun.* **60**, 400–403 (2024). 10.1039/d3cc05451a 38079184

[10] Badri, Z. & Foroutan-Nejad, C. Classical versus collective interactions in asymmetric trigonal bipyramidal alkaline metal‒boron halide complexes. *Chemistry***n/a**, e202400156 (2024). 10.1002/chem.202400156 38642012

[11] Pendás, A. M., Blanco, M. A. & Francisco, E. Steric repulsions, rotation barriers, and stereoelectronic effects: a real space perspective. *J. Comput. Chem.* **30**, 98–109 (2009). 18536054 10.1002/jcc.21034

[12] Schneider, W. B. et al. Decomposition of intermolecular interaction energies within the local pair natural orbital coupled cluster framework. *J. Chem. Theory Comput.* **12**, 4778–4792 (2016). 27564403 10.1021/acs.jctc.6b00523

[13] Xiao, M. & Lu, T. Generalized charge decomposition analysis (GCDA) method. *J. Adv. Phys. Chem.* **4**, 111–124 (2015).

[14] Dapprich, S. & Frenking, G. Investigation of donor-acceptor interactions: a charge decomposition analysis using fragment molecular orbitals. *J. Phys. Chem.* **99**, 9352–9362 (1995).

[15] Pearson, R. G. *Chemical Hardness: Applications from Molecules to Solids* (Wiley-VCH, 1997).

