# Supplementary material for: Reply to: An approach to the resolution of the dispute on collective atomic interactions
Source: Nat Commun. 2024 Nov 30;15:10403. doi: 10.1038/s41467-024-54553-y (PMC11608374; doi:10.1038/s41467-024-54553-y)
Supplement: Supplementary file 1 — Supplementary Information [file 41467_2024_54553_MOESM1_ESM.pdf]

Supplementary Material

Reply to: An approach to the resolution of the dispute on collective atomic interactions

Nuno A. G. Bandeira[1]*, Angel Martin-Pendas[2]*, Cina Foroutan-Nejad[3]*

1. Biosystems and Integrative Sciences Institute (BioISI)- Departamento de Química e Bioquímica, Faculdade de Ciências Universidade de Lisboa 8.5.53 - C8 Campo Grande,1749-016 Lisboa, Portugal
2. Department of Química Física y Analítica. Universidad de Oviedo. C/ Julián Clavería 8. 33006 Oviedo, Spain.
3. Institute of Organic Chemisrty, Polish Academy of Sciences, Kasprzaka 44/52, 01-224 Warsaw, POLAND

Corresponding authors' emails

NAGB: nuno.bandeira@ciencias.ulisboa.pt
AMP: ampendas@uniovi.es
CFN: cina.foroutan-nejad@icho.edu.pl

Computational Details

Geometry optimizations were performed at the CCSD/def2-TZVPP level in ORCA[1,2] version 5.0.4. The Resolution of Identity[3] density fitting procedure was applied with the corresponding correlated auxiliary basis set[4] def2-TZVPP/C. The LED[5] analysis was performed using a DLPNO-CCSD(T) formalism[6–8] with the *TightPNO* setting. While the EDA by Ziegler and Rauk[9] relies on a density functional formulation with all its shortcomings, the recently proposed Local Energy Decomposition (LED) scheme proposed by Schneider and colleagues[5] allows for a partitioning of the energy terms of a coupled-cluster calculation employing localized pair natural orbitals.[6] A previous charge assignment to each fragment is not required except if it is necessary to estimate the distortion energies of either fragment with respect to their equilibrium geometries. In this aspect it is analogous to the better known symmetry adapted perturbation theory (SAPT) method.[10] The generalized charge decomposition analysis[11] (GCDA) is an extension of Dapprich and Frenking's[13] charge decomposition analysis scheme for correlated methods. Its use can assign charge transfers to explicit natural orbitals of any post-HF wavefunction. The GCDA analysis[11] was carried out with the MultiWfn[12] 3.8 program on the CCSD natural orbitals. The GCDA analysis consists of relaxing the superposition of two fragment wavefunctions as with any conventional EDA scheme. The outcome is that electron traffic can be mapped upon changing from the individual fragment to the adduct species. In both isomers the resulting $CF_3 \rightarrow$ Li electron donation overcomes the Li $\rightarrow CF_3$ charge transfer value. The subtraction of both values results in a net $CF_3 \rightarrow$ Li charge transfer value. Furthermore, electron reorganizations in the overlap region of the fragments *r* allow both fragments to either accumulate charge in the overlap

(r>0) or deplete it (r<0) in the case of electron repulsion in the presence of each other. These values bear no relevance to the analysis at hand.

1. General notes on GCDA

CDA whether in its generalised form or not brings the sum of two terms: one of polarisation (PL) or readjustment of the MOs when one fragment is brought to the presence of another fragment. This takes place through coefficient mixing between occupied and virtual basis functions within each fragment individually, and the second more crucial term of inter-fragment charge transfer (CT), i.e.

$$PL(1) + CT(1\rightarrow2) - PL(2) - CT(2\rightarrow1) \quad (1)$$

The dissection of both terms has been made possible through Gorelsky's extended CDA scheme (ECDA)[14] which provides the actual CT(1→2) – CT(2→1) difference. As such the numbers in (generalized) CDA and ECDA will differ depending on the partitioning of the fragments. Unfortunately, ECDA was only devised for single determinant methods such as Hartree-Fock or DFT which limits its applicability in our case to CCSD natural orbitals. However, to a good approximation the vast majority of polarisation effects can already be extracted from the HF wavefunction. Considering our case of $LiCF_3$, if one partitions the fragments as neutral $Li^{\bullet}$ and $CF_3^{\bullet}$ then it is necessary to estimate the effect of MO polarisation from ECDA for the (G)CDA numbers to make sense.

Let us take the values of the GCDA analysis at the CCSD/def2-TZVPP level for the neutral fragments:

Table S 1 – GCDA with neutral fragments.

	i-$LiCF_3$	p-$LiCF_3$
PL(Li) + CT(Li→CF_3) – PL(CF_3) – CT(CF_3→Li)	-0.1363	-0.0602
Overlap population r	0.0242	0.0165

By performing an ECDA on the HF wavefunction one obtains

Table S 2 – ECDA with neutral fragments.

	i-$LiCF_3$	p-$LiCF_3$
PL(Li) + CT(Li→CF_3) – PL(CF_3) – CT(CF_3→Li)	-0.1160 e^-	-0.0515
CT(Li→CF_3) – CT(CF_3→Li)	0.806	0.871

Since the CDA figures don't differ much from the GCDA one may confident that the polarisation term ΔPL may be reasonably approximated by the HF wavefunction. By subtracting the values of the second from the first row of the preceding Table S 2 one gets ΔPL(i-$LiCF_3$)=0.922 and ΔPL(p-$LiCF_3$)=0.927. So, the actual CT terms in Table S 1 may be approximated in the following way

	i-$LiCF_3$	p-$LiCF_3$
PL(Li) + CT(Li→CF_3) – PL(CF_3) – CT(CF_3→Li)	-0.1363	-0.0602
CT(Li→CF_3) – CT(CF_3→Li)	0.786	0.867

It is easy to demonstrate in this manner that for the ionic partitioning (Li^+ + CF_3^-) the polarisation is minimal. Since the ECDA yields ΔPL(i-$LiCF_3$)=0.029 and ΔPL(p-$LiCF_3$)= 0.021 the ionic partitioning is a good reflection of the bound fragments such

that to a good approximation the outcome of the GCDA with ionic partitioning can be thought of as almost exclusively charge transfer terms.

Table S 3 – Combined GCDA/ECDA with approximate net charge transfer in the ionic case.

	i-$LiCF_3$	p-$LiCF_3$
PL(Li) + CT(Li→CF_3) – PL(CF_3) – CT(CF_3→Li)	-0.1993	-0.1239
CT(Li→CF_3) – CT(CF_3→Li)	-0.1703	-0.1029

In the first row of the preceding Table S 3 are the same GCDA values listed in Table 2 of the main text as they are a good representation of the overall charge transfer processes.
Therefore, when performing GCDA some caution should be exercised to consider the MO polarization effects and how fragment charge assignment might affect the overall analysis. In highly ionic bonds such as $LiCF_3$ it is simple to approximate the GCDA figures as almost exclusively CT but in intermediate cases where fragment bonding is between ionicity and covalency the GCDA may be more nuanced and it may be required to perform both types of fragmentation and crosscheck with ECDA.

1. The geometries

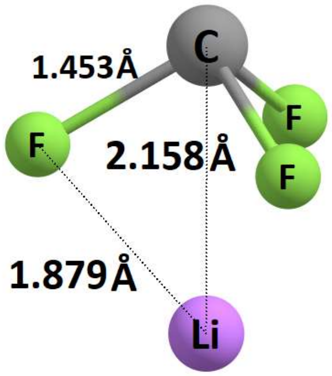

Figure S1 - i-$LiCF_3$ optimized coordinates

C	0.00000000000000	0.00000000000000	0.76067385898707
Li	0.00000000000000	0.00000000000000	-1.39759390349197
F	-0.62223229668702	1.07773795197219	0.00990008709415
F	-0.62223229668702	-1.07773795197219	0.00990008709415
F	1.24446459337404	0.00000000000000	0.00990008709415

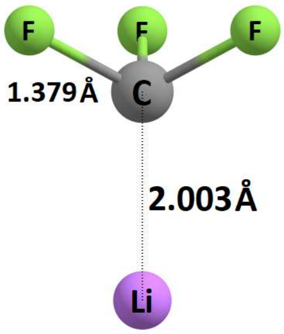

Figure S2 – p-$LiCF_3$ optimized coordinates

C	0.00000000000000	0.00000000000000	-0.25603577414164
Li	0.00000000000000	0.00000000000000	-2.25854893134143
F	0.62436807821069	1.08143723408506	0.32901417369646
F	0.62436807821069	-1.08143723408506	0.32901417369646
F	-1.24873615642139	-0.00000000000000	0.32901417369646

References:

1. Neese, F. Software update: The ORCA program system—Version 5.0. *WIREs Comput. Mol. Sci.* **12**, e1606 (2022).
2. Neese, F., Wennmohs, F., Becker, U. & Riplinger, C. The ORCA quantum chemistry program package. *J. Chem. Phys.* **152**, 224108 (2020).
3. Neese, F. An improvement of the resolution of the identity approximation for the formation of the Coulomb matrix. *J. Comput. Chem.* **24**, 1740–1747 (2003).
4. Hellweg, A., Hättig, C., Höfener, S. & Klopper, W. Optimized accurate auxiliary basis sets for RI-MP2 and RI-CC2 calculations for the atoms Rb to Rn. *Theor. Chem. Acc.* **117**, 587–597 (2007).
5. Schneider, W. B. *et al.* Decomposition of Intermolecular Interaction Energies within the Local Pair Natural Orbital Coupled Cluster Framework. *J. Chem. Theory Comput.* **12**, 4778–4792 (2016).

6. Riplinger, C. & Neese, F. An efficient and near linear scaling pair natural orbital based local coupled cluster method. *J. Chem. Phys.* **138**, 034106 (2013).

7. Riplinger, C., Pinski, P., Becker, U., Valeev, E. F. & Neese, F. Sparse maps—A systematic infrastructure for reduced-scaling electronic structure methods. II. Linear scaling domain based pair natural orbital coupled cluster theory. *J. Chem. Phys.* **144**, 024109 (2016).

8. Neese, F., Hansen, A. & Liakos, D. G. Efficient and accurate approximations to the local coupled cluster singles doubles method using a truncated pair natural orbital basis. *J. Chem. Phys.* **131**, 064103 (2009).

9. Ziegler, T. & Rauk, A. On the calculation of bonding energies by the Hartree Fock Slater method. *Theor. Chim. Acta* **46**, 1–10 (1977).

10. Szalewicz, K. Symmetry-adapted perturbation theory of intermolecular forces. *WIREs Comput. Mol. Sci.* **2**, 254–272 (2012).

11. Xiao, M. & Lu, T. Generalized Charge Decomposition Analysis (GCDA) Method. *J. Adv. Phys. Chem.* **4**, 111–124 (2015).

12. Lu, T. & Chen, F. Multiwfn: A multifunctional wavefunction analyzer. *J. Comput. Chem.* **33**, 580–592 (2012).

13. Dapprich, S. & Frenking, G. Investigation of Donor-Acceptor Interactions: A Charge Decomposition Analysis Using Fragment Molecular Orbitals. *J. Phys. Chem.* **99**, 9352–9362 (1995).

14. Gorelsky, S. I., Ghosh, S. & Solomon, E. I. Mechanism of N2O Reduction by the μ4-S Tetranuclear CuZ Cluster of Nitrous Oxide Reductase. *J. Am. Chem. Soc.* **128**, 278–290 (2006).
